# Placental morphology in association with autism-related traits in the EARLI study

**DOI:** 10.1186/s12884-022-04851-4

**Published:** 2022-06-28

**Authors:** Caichen Zhong, Ruchit Shah, Juliette Rando, Bo Park, Theresa Girardi, Cheryl K. Walker, Lisa A. Croen, M. Daniele Fallin, Irva Hertz-Picciotto, Brian K. Lee, Rebecca J. Schmidt, Heather E. Volk, Craig J. Newschaffer, Carolyn M. Salafia, Kristen Lyall

**Affiliations:** 1 Department of Epidemiology and Biostatistics, Dornsife School of Public Health, Drexel University, Philadelphia, PA 19104 USA; 2 Placental Analytics, New Rochelle, NY USA; 3 AJ Drexel Autism Institute, Drexel University, Philadelphia, PA USA; 4 Department of Public Health, California State University Fullerton, Fullerton, CA USA; 5 Department of Obstetrics and Gynecology, University of California Davis Health, Sacramento, CA USA; 6 Division of Research, Kaiser Permanente Northern California, Oakland, CA USA; 7 Department of Mental Health, Johns Hopkins University, Baltimore, MD USA; 8 Department of Public Health Sciences and the MIND Institute, School of Medicine, University of California Davis, Davis, CA USA; 9 Department of Health and Human Development, Pennsylvania State University, University Park, PA USA

**Keywords:** Autism spectrum disorder, Placental morphology, Placental thickness, Umbilical cord, Autism-related traits, Cluster analysis

## Abstract

**Background:**

In prior work we observed differences in morphology features in placentas from an autism-enriched cohort as compared to those from a general population sample. Here we sought to examine whether these differences associate with ASD-related outcomes in the child.

**Methods:**

Participants (*n* = 101) were drawn from the Early Autism Risk Longitudinal Investigation (EARLI), a cohort following younger siblings of children with autism spectrum disorder (ASD). ASD-related outcomes, including the Social Responsiveness Scale (SRS), Mullen Scales of Early Learning (MSEL) Early Learning Composite, and ASD diagnosis, were assessed at age 3. Crude and adjusted linear regression was used to examine associations between placental morphological features (parametrized continuously and in quartiles) and SRS and MSEL scores; comparisons by ASD case status were explored as secondary analyses due to the small number of cases (*n* = 20).

**Results:**

In adjusted analyses, we observed a modest positive association between umbilical cord eccentricity, defined as the ratio of the maximum:minimum radius from the cord insertion point, and SRS scores (Beta = 1.68, 95%CI = 0.45, 2.9). Positive associations were also suggested between placental maximum thickness and cord centrality and SRS scores, though these were estimated with little precision. Associations between other placental morphological features and outcomes were not observed.

**Conclusions:**

Our analyses suggested a potential association between umbilical cord features and ASD-related traits, of interest as non-central cord insertion may reflect reduced placenta efficiency. Future studies with larger sample sizes are needed to further examine these and other placental features in association with ASD-related outcomes.

**Supplementary Information:**

The online version contains supplementary material available at 10.1186/s12884-022-04851-4.

## Background

The placenta is critical to the exchange of oxygen and nutrients between the mother and fetus, as well as in providing protection to the developing fetus and supporting its growth [1]. Placental morphologic features are important indicators of placental function. Specifically, abnormal placental shape has been associated with lower birthweights than predicted by allometric fetoplacental scaling, which may be due to reduced placental efficiency [2–6]. Eccentric umbilical cord insertion, independent of placental shape, has also been associated with reductions in vascular efficiency, as well as reduced fetoplacental ratio (fetal weight divided by placental weight) [4, 5, 7]. Studies have also shown that placental thickness impacts functional efficiency, even after adjusting for other placental measures or maternal characteristics [2, 8, 9]. Furthermore, placental morphological features and complications have been linked not only with birthweight [4, 10, 11], fetal growth restriction (FGR) [12] and preterm delivery [13], but also Apgar scores [14], motor development and cerebral palsy [15], and longer-term child outcomes including body mass index (BMI), cardiovascular disease, hypertension, and asthma [11, 16–18]. Placental morphological features and complications like placental inflammation and abruption have also been associated with delayed neurodevelopment as indicated by lower scores on cognitive assessments, as well as antisocial behavior, and attention deficit hyperactivity disorder [4, 19–23]. Yet it is not clear how these features may relate to other specific neurodevelopmental conditions like autism spectrum disorder (ASD).

ASD is a complex developmental condition defined by social communication and behavioral challenges that currently affects an estimated 1 in 54 children in the United States [24]. Studies have supported that the pathophysiology of ASD originates during fetal development [25]. Several meta-analyses have shown strong associations between ASD and advanced parental age, low birthweight, small for gestational age, as well as other pregnancy complications which may be indicators of placental abnormalities, such as antepartum hemorrhage, gestational hypertension, gestational diabetes, and preeclampsia [26–29]. Some work has suggested that placental abnormalities, such as placenta abruptia and previa, and umbilical cord knots, are asssociated with ASD-related outcomes, including ASD diagnosis and ASD severity according to continuous trait measures, though most findings have not been replicated [28, 30].

Only a handful of studies have examined potential associations between additional placental features and ASD. Two studies suggested an association between ASD and placental inflammation [31], though one of these was an additive association between chorioamnionitis and preterm birth [32]. Several studies have also suggested differences in placental features according to familial increased probability of autism, defined as having a prior child with ASD. In prior work comparing placental features from the Early Autism Risk Longitudinal Investigation (EARLI), a high autism probability cohort, to the National Children’s Study (NCS), a general population cohort, differences were observed. Placentas from EARLI were more regularly shaped relative to those from the NCS, which was suggested to reflect reduced capacity to respond to changes [33]. Additional studies have also suggested differences in placental vasculature in placentas from these high autism probability families [34, 35]. Whether the observed differences translate to associations with ASD-related outcomes, however, is not yet known.

The present study therefore sought to extend our prior work by examining associations with autism-related outcomes in children from EARLI. We focused on those placental morphological features identified in our prior work as associated with familial autism status, and explored associations with a broader list of placental morphological features. Our primary analyses of ASD-related outcomes focused on quantitatively assessed ASD-related traits, including the Social Responsiveness Scale (SRS), as a widely used measure of social communication and ASD-related phenotype, as well as the Mullen Scales of Early Learning (MSEL), as a measure of early cognitive development. As one of the few ASD studies with prospectively collected placentas, our study provides novel information about the relationship between placental features and ASD-related outcomes.

## Methods

### Study participants

**Fig. 1:**
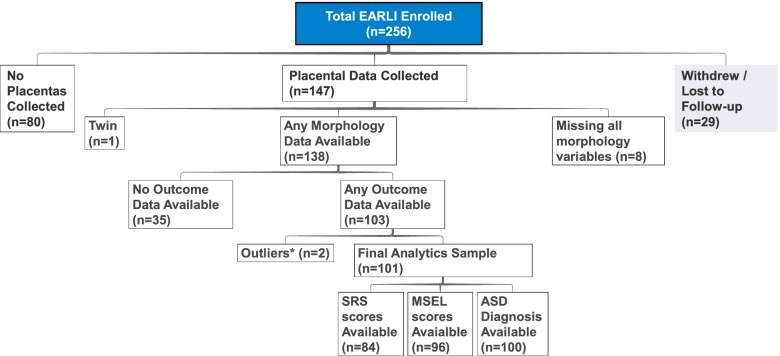
Analysis Population from the EARLI study. *Outliers are those with out-of-range placental weight and eccentricity measures. Flow chart showing the selection of the study population. 29 mothers withdrew from the study, while 80 did not have placentas collected or measures taken; of remaining 147 participants, 8 did not have placental morphology measurements available; 1 twin was excluded, and 35 children with no outcome data were excluded. Finally, two individuals were excluded as outliers, due to placental weight and eccentricity values out of the plausible range. This yielded a final analytic sample of 101 individuals with any outcome information available, which “floated” across analyses of individual outcomes (range 84–100)

Participants were drawn from EARLI, a multisite prospective cohort enrolling families with a higher probability of ASD due to already having a child on the spectrum (ASD-enriched cohort). Details on the EARLI study are provided elsewhere [36]. Briefly, from 2009 to 2012, EARLI enrolled eligible pregnant mothers by the 28th week of pregnancy at four U.S. sites (Philadelphia, Baltimore, San Francisco Bay Area, and Sacramento) [36]. Additionally, eligible women were those aged 18 years or older and able to communicate in English or Spanish. The children from these pregnancies were then followed from birth to 36 months. In order to be included in the present analyses, individuals must have had at least one placental morphology feature measured, and at least one of the ASD-related outcome measures of interest (described below). Following exclusions and removal of outliers (those with implausible values of placental weight and eccentricity measures) and one child from a twin birth (randomly selected), 101 mother-child pairs were included in the final analysis (Fig. 1). The EARLI Study was approved by the Drexel University Institutional Review Board and local IRBs at all EARLI Study sites.

### Placental features

Placental tissue was collected at the time of childbirth and preserved in a sealed bag with formalin [36]. Placental gross morphology measures were collected by a trained pathologist following a standard protocol. Features that broadly capture placental shape and cord irregularity characteristics were measured and calculated as previously described [33]. After trimming the extraplacental membranes and umbilical cord, 2D digital images of the fetal placental chorionic surface shape were taken, to measure the umbilical cord insertion site, perimeter, area, and the sliced placental disk site, according to central slice along the visibly longest diameter through the insertion point. The placental perimeter was calculated from the traced chorionic disk shape by counting the edge pixels. The umbilical distance from centroid (also known as cord centrality) was calculated as the distance between umbilical cord and centroid of the placenta as determined from the traced 2D fetal surface image. Placental eccentricity was captured according to 1) cord eccentricity, defined as the ratio of the maximum:minimum radius from the umbilical insertion point, and 2) shape eccentricity (also known as placental ellipsivity), defined as the ratio of the maximum:minimum radius from the geometric center. For both measures, a value of 1 indicates low eccentricity (a centrally inserted umbilical cord for the former and a round placenta for the latter) [6]. Following our prior work [33] [37], variables examined here included placental weight at delivery (grams), perimeter (cm), area (cm^2^), umbilical cord distance from center (also called cord centrality; cm), radius (cm, measured as radius from geometric center to the perimeter of the placental disk measured per degree, as median, minimum, and maximum values), the aforementioned eccentricity measures, and thickness (cm) of the placenta disk slice (including maximum, mean, standard deviation). In addition, the fetoplacental ratio, the ratio of birthweight to placental weight, was calculated. These gross variables are also listed and defined in eTable 1. We also decided to use the k-means clustering analysis of additional placental variables as the exploratory analysis to gain a more complete understanding of the association between placental morphology features and autism-related outcomes.

### Outcomes

Due to the small number of ASD cases (*n* = 20) in our analytic sample, we focused analyses here on the continuous SRS and MSEL scores as the primary outcomes, while analyzed dichotomous ASD diagnosis as a secondary outcome for comparison purposes.

ASD-related traits were captured according to the preschool version of the SRS, collected at 36 months during an in-person clinic visit [38]. The SRS is a widely used quantitative measure of social communication and autistic phenotype [38]. The measure consists of 65 items completed by a parent or caregiver, which are summed to yield a total continuous raw score; higher scores indicate greater autism-related traits [38].

Cognitive development was measured according to MSEL Early Learning Composite (ELC) at 36 months during the in-person clinic visit [39]. Cognitive development is of interest to examine within the context of ASD given evidence of co-occurrence of intellectual disability and ASD [40]. The MSEL assesses broad intellectual development and school readiness, with ELC providing a summary score of cognitive functioning. Higher ELC score indicates greater cognitive and motor development.

ASD diagnosis, evaluated at 36 months, was defined according to best-estimate clinical consideration of DSM-5 criteria, Autism Diagnostic Observation Schedule (ADOS) scores, and the Autism Diagnostic Interview-Revised (ADI-R).

### Statistical analysis

Basic descriptive statistics of placental features were examined in the study population. To examine associations between our primary placental morphology features and continuous ASD-related outcomes (SRS and MSEL ELC scores), we used crude and adjusted linear regression. Raw total SRS scores were used in primary analyses; T scores were used in secondary analyses to provide clinical context. Placental variables were examined as continuous values as well as in quartiles, using the interquartile range as the referent group. In addition, given certain measures may differ based on overall placental size, we also examined associations with the radii measures and cord distance from center standardized for overall placental area, similar to approaches used in prior work [9, 21, 41–43]. Covariates in all models were selected on the basis of a priori associations with ASD and potential relevance to placental features. Multivariable models included adjustment for maternal age (continuous), maternal race/ethnicity (non-Hispanic white vs. non-white), maternal education level (some college or below, college degree, graduate school or above), maternal pre-pregnancy body mass index (BMI; continuous), and the child’s sex. Models with and without adjustment for gestational age were also examined. Furthermore, interaction by sex was explored in separate adjusted models using an interaction term between continuously parameterized placental factors and child’s sex. Covariate missingness was low (< 10%). In order to maintain sample size, missing covariate values were imputed with the median (categorical covariates) or mean (continuous covariates). Analyses were performed using SAS 9.4.

In secondary analyses, given the small number of cases (*n* = 20), we conducted exploratory secondary analyses of associations with ASD diagnosis using logistic regression, for purposes of comparison to primary results only.

We also conducted a series of exploratory analyses using k-means clustering to further examine a larger set of 34 placental features, using two different variables selection strategies to ensure consistency (See Appendix). In the first approach, principal components analysis (PCA) was used as a data reduction approach prior to k-means clustering. In a second approach, placental variables for clustering were selected according to suggestions of their importance in the literature [6, 19, 33, 44]. We then utilized clusters according to each of the data-driven and literature-driven approaches in multivariable regression models to explore associations with child outcomes.

## Results

Basic characteristics of the study population are shown in Table 1. The majority of mothers in the analytic sample were non-Hispanic white, US-born, college-educated, and had household incomes of $100,000 or more, with a mean age of 34.6 years. Sociodemographic characteristics of participants included in this analysis were broadly comparable to EARLI-enrolled participants overall (eTable 3). Basic descriptive statistics of placental morphology variables are shown in Table 2. Placenta radius and weight in our sample were comparable to reference ranges for full term pregnancies, while placenta perimeter, area, and thickness were somewhat smaller on average than available reference mean values for pregnancies of 37–40 weeks [45]. A Pearson’s r correlation coefficient was computed to assess the relationship between individual placental morphology variables, ranges from − 0.73 to 0.94 (eTable 2).

**Table 1:** Basic characteristics of the EARLI analytic sample (*n* = 101)

Maternal characteristics	Mean (SD) or n(%)^a^
Maternal Age (years)	34.6 (4.18)
Maternal Race/Ethnicity	
Non-Hispanic White	62 (61.4%)
Hispanic	10 (9.9%)
Non-Hispanic Black	15 (14.9%)
Non-Hispanic Asian	10 (9.9%)
Other	3 (3.0%)
Missing	1 (1.0%)
Maternal Education Level	
Some college or below	33 (32.7%)
College degree	31 (30.7%)
Graduate School or above	35 (34.7%)
Missing	2 (2.0%)
Maternal Birthplace	
US	84 (83.2%)
Outside U.S.	16 (15.8%)
Missing	1 (1.0%)
Maternal Pre-pregnancy BMI	27.1 (6.67)
Household Income	
Less than $50,000	25 (24.8%)
$50,000 to $99,999	26 (25.7%)
$100,000 or more	47 (46.5%)
Missing	3 (3.0%)
**Child characteristics**	**Mean (SD) or n(%)**^a^
Child Sex	
Female	46 (45.5%)
Male	55 (54.5%)
Child Birthweight	
Low birthweight (< 2500 g)	4 (4.0%)
Normal birthweight	96 (95.0%)
Missing	1 (1.0%)
Gestational Age (weeks)	39.4 (1.32)
Delivery Mode	
Vaginal Birth	61 (60.4%)
C-section	38 (37.6%)
Missing	2 (2.0%)
SRS Total Raw Score at 36mos	32.9 (27.1)
MSEL ELC Score at 36mos	99.2 (20.7)
ASD Diagnosis at 36mos	
No ASD	80 (79.2%)
ASD	20 (19.8%)
Missing	1 (1.0%)

^a^Maternal Pre-pregnancy BMI missing *n* = 4. SRS Total Raw Score at 36mos missing *n* = 17. MSEL ELC Score at 36mos missing *n* = 5

**Table 2:** Descriptive statistics of placental morphology features

	N^a^	Mean	Median	SD	Min	Max	Reference ranges^b^
Perimeter (cm)	101	62.43	61.99	7.55	45.92	92.43	63–69
Area (cm2)	101	278.20	277.38	52.07	160.93	439.80	314–380
Radius (cm)							
Minimum	101	5.27	5.66	2.04	0.67	8.95	10–11
Maximum	101	13.73	13.34	2.30	9.65	19.04	
Median	101	10.14	9.93	1.19	7.40	13.88	
Umbilical cord distance from center (cm)	101	3.52	3.21	2.07	0.23	8.95	
Eccentricity							
Eccentricity from geometric center	101	1.41	1.35	0.25	1.11	2.64	
Eccentricity from cord insertion point	101	3.80	2.40	4.29	1.23	28.01	
Thickness of traced central slice (cm)							
Maximum	98	2.38	2.32	0.33	1.69	3.49	2.0–2.5
Mean	98	1.67	1.62	0.26	1.20	2.65	
Placental weight (g)	94	465.29	461.50	80.67	284.00	692.00	414–470
Birthweight: Placental Weight	93	7.55	7.55	1.16	4.56	10.88	6.04–7.23

^a^Two outliers were removed in the analyses

^b^Reference: Benirschke, K. and P. Kaufmann, Pathology of the human placenta. 2000, Springer: New York. p. 921–922

In adjusted analyses of associations with SRS scores, an increase in umbilical cord eccentricity was associated with a modest increase in raw SRS score (ß = 1.68, 95%CI = 0.45, 2.90). Increasing placental thickness was associated with a larger increase in raw SRS score (ß for maximum thickness of traced central slice = 17.12, 95%CI = − 0.00, 34.24) (Table 3). Associations were not observed with other features. In analyses utilizing standardized versions of relevant placental variables (radius/area and cord centrality/area), results overall were consistent with primary analyses, indicating no associations with these parameters (eTable 4). In secondary analyses examining associations with highest and lowest quartiles (vs interquartile) of placental features, greater cord distance from the center was associated with higher SRS score (adjusted ß for Q4 vs IQR = 13.55, 95% CI = -0.70, 27.81, consistent with nearly a 1/2 SD unit increase in SRS score). Across analyses, estimates were similar without (model 1) and with (model 2) adjustment for gestational age. Exploratory analyses showed significant interactions by child sex with placental perimeter(*p* = 0.02) and area (*p* = 0.02), but not with other features (interaction results are not shown).

**Table 3:** Crude and adjusted associations (ß estimates and 95% confidence intervals) between placental morphological features and child raw total SRS scores

Variable^a^	n	Crude	Model 1^b^	Model 2^c^
		ß (95% CI)	ß (95% CI)	ß (95% CI)
Perimeter (cm)	84	−0.22 (− 0.97, 0.53)	0.11 (− 0.62, 0.84)	0.10 (− 0.64, 0.84)
Area (cm^2^)	84	− 0.02 (− 0.13, 0.09)	0.03 (− 0.07, 0.14)	0.03 (− 0.08, 0.14)
Radius (cm)				
Minimum	84	−2.3 (−5.22, 0.61)	− 1.01 (−3.92, 1.89)	− 1.04 (−3.97, 1.88)
Maximum	84	1.73 (− 0.76, 4.23)	1.16 (− 1.19, 3.51)	1.14 (− 1.23, 3.51)
Median	84	1.96 (−3.09, 7.00)	2.67 (− 2.03, 7.38)	2.63 (− 2.15, 7.41)
Umbilical cord centrality/distance from center (cm)	84	2.3 (− 0.50, 5.10)	1.12 (−1.64, 3.89)	1.12 (− 1.67, 3.90)
Eccentricity				
Eccentricity from Geometric center	84	0.31 (−23.10, 23.71)	2.96 (−19.42, 25.34)	3.05 (−19.47, 25.58)
Eccentricity from cord insertion point	84	1.95 (0.69, 3.21)	1.68 (0.47, 2.89)	1.68 (0.45, 2.9)
Thickness of traced central slice (cm)				
Maximum	81	17.00 (−1.08, 35.09)	17.00 (0.20, 33.79)	17.12 (−0.003, 34.24)
Mean	81	13.37 (−9.88, 36.62)	12.41 (−9.32, 34.14)	12.29 (−9.70, 34.29)
Thickness of traced central slice (cm) (Z score)				
Maximum	81	5.69 (− 0.36, 11.74)	5.69 (0.07, 11.30)	5.73 (− 0.00, 11.46)
Mean	81	3.48 (−2.57, 9.52)	3.23 (−2.42, 8.88)	3.20 (− 2.52, 8.91)
Placental weight (g)	79	0.04 (−0.03, 0.11)	0.06 (−0.01, 0.13)	0.06 (− 0.02, 0.13)
Birthweight: Placental Weight	79	−2.99 (−9.31, 3.34)	−5.82 (−12.15, 0.51)	−6.05 (− 12.42, 0.31)

^a^Placental eccentricity is the ratio of the maximum radius divided by the minimum radius from the geometric center or the umbilical insertion point; a round placenta with a centrally inserted umbilical cord would have a cord eccentricity of 1

^b^Model 1 adjusted for child sex, maternal age, maternal education level, maternal pre-pregnancy BMI

^c^Model 2 adjusted for all covariates from Model 1 and additionally adjusted for child gestational age. Additional adjustment for maternal race/ethnicity did not materially alter results

No associations between the placental features and MSEL ELC scores were observed (Table 4; eTable 5). Most placental features examined were associated with small decreases in ELC scores with CIs overlapping the null. Similar null results were observed when parameterizing placental features in quartiles (eTable 6). No significant interactions by child sex were observed.

**Table 4:** Crude and adjusted associations (ß estimates and 95% confidence intervals) between individual placental variables and child MSEL ELC scores

Variable^a^	n	Crude	Model 1^b^	Model 2^c^
		ß (95% CI)	ß (95% CI)	ß (95% CI)
Perimeter (cm)	96	0.02 (−0.58, 0.61)	− 0.12 (− 0.67, 0.44)	−0.1 (− 0.66, 0.46)
Area (cm^2^)	96	−0.01 (− 0.10, 0.07)	−0.03 (− 0.11, 0.05)	−0.03 (− 0.11, 0.05)
Radius (cm)				
Minimum	96	0.1 (−1.96, 2.17)	−0.39 (− 2.36, 1.57)	− 0.37 (− 2.34, 1.60)
Maximum	96	− 0.33 (− 2.15, 1.49)	0.16 (− 1.55, 1.87)	0.18 (− 1.53, 1.90)
Median	96	−0.66 (−4.25, 2.93)	−1.53 (− 4.88, 1.82)	−1.36 (− 4.75, 2.03)
Umbilical distance from center (cm)	96	− 0.34 (− 2.39, 1.71)	0.28 (− 1.68, 2.24)	0.28 (− 1.68, 2.24)
Eccentricity				
Eccentricity from Geometric center	96	4.56 (−13.56, 22.69)	4.6 (− 12.19, 21.39)	4.91 (− 11.91, 21.74)
Eccentricity from Umbilical cord	96	−0.51 (− 1.48, 0.45)	− 0.41 (− 1.32, 0.50)	−0.39 (− 1.30, 0.52)
Thickness of traced central slice (cm)				
Maximum	93	− 8.02 (− 20.85, 4.8)	−4.96 (− 17.00, 7.08)	−4.50 (− 16.77, 7.77)
Mean	93	−8.63 (− 25.17, 7.92)	−5.63 (− 21.17, 9.9)	−5.24 (− 20.92, 10.45)
Thickness of traced central slice (cm) (Z score)				
Maximum	93	− 2.68 (− 6.97, 1.61)	−1.66 (− 5.69, 2.37)	−1.51 (− 5.61, 2.60)
Mean	93	−2.24 (− 6.54, 2.06)	−1.46 (− 5.50, 2.57)	−1.36 (− 5.44, 2.72)
Placental weight (g)	89	− 0.03 (− 0.09, 0.03)	−0.03 (− 0.09, 0.02)	−0.03 (− 0.09, 0.03)
Birthweight: Placental Weight	88	−1.08 (− 5.56, 3.40)	− 0.09(− 4.54, 4.36)	0.07(−4.49, 4.62)

^a^Placental eccentricity is the ratio of the maximum radius divided by the minimum radius from the geometric center or the umbilical insertion point; a round placenta with a centrally inserted umbilical cord would have a cord eccentricity of 1

^b^Model 1 adjusted for child sex, maternal age, maternal education level, maternal pre-pregnancy BMI

^c^Model 2 adjusted for all covariates from Model 1 and additionally adjusted for child gestational age. Additional adjustment for maternal race/ethnicity did not materially alter result

In exploratory analyses, overall, associations were not observed with ASD diagnosis, though we lacked statistical precision due to a small number of cases (eTable 7). However, there was a small increase in odds of ASD with increased cord eccentricity, consistent with our analyses of SRS scores (adjusted OR 1.13, 95% CI = 1.00, 1.27). Other features yielded generally null and/or imprecise estimates.

In exploratory cluster analyses (Appendix A), 19 placental measures identified through PCA were used in our first “data-driven” cluster analysis. Two clusters were identified. The first contained individuals generally characterized by smaller, more uniform, but thicker placentas, while the second cluster contained those characterized by larger, thinner placentas and a less centrally inserted umbilical cord (eFigure 1). In literature-driven” analyses including 15 placental measures selected based on literature support for prior associations with birth outcomes, results were similar to the data driven approach, with the exception that lower values for eccentricity were grouped in the cluster characterized by smaller and thicker placentas rather than the second cluster that included larger, thinner placentas (eFigure 2). However, when examining these clusters for associations with SRS and MSEL scores, no associations were observed (eTable 8).

## Discussion

In this study examining placental morphology features in association with child ASD-related outcomes in a high-autism probability cohort, overall, we did not see evidence for strong associations. However, our results did suggest a modest positive association between umbilical cord eccentricity measures and SRS scores. Specifically, increased ASD-related traits were observed with greater umbilical cord eccentricity, though the effect size was small. Increased placental thickness, and in some analyses, an additional measure capturing cord non-centrality, also demonstrated positive associations with SRS scores, but these were estimated with low precision.

Previous studies have suggested differences between placentas from high autism probability pregnancies and those from the general population. Prior analyses in EARLI suggested placentas in the high autism probability group were rounder, thicker, and more regular in perimeter with reduced variability than in those drawn from the general population [33]. Here, we extended this work to examine relationships with child outcomes, and generally did not see evidence that the same placental features associate with ASD-related outcomes within this high autism probability setting. Park and colleagues reported no differences in cord insertion measures [33], while our work did suggest associations with cord eccentricity and child ASD-related traits. Although both studies reported some positive association between placental thickness and ASD-study population or ASD-related traits, this association was estimated with little precision in our study. While our study may have lacked statistical power to detect modest associations due to our small sample size, the discrepancies in findings may mean certain features are linked with genetic factors differing between high autism probability and low autism probability families, but not necessarily with later child developmental outcomes according to measures examined here.

The positive association between umbilical cord eccentricity and ASD-related traits we observed is consistent with some prior work suggesting relationships with offspring outcomes. Eccentric cord insertion (or marginal cord insertion) has been associated with preterm birth, low birthweight, and small for gestational age [46–48], which are risk factors for various child neurodevelopmental outcomes, including ASD. In addition, some work linking cord eccentricity to neurodevelopmental outcomes comes from other study populations that could be considered different types of high-autism probability populations. A multicenter retrospective cohort study of severe growth-restricted fetuses suggested that abnormal cord insertion was associated with poor long term neurological prognoses, such as cerebral palsy and developmental disorders [15].

While we cannot rule out the potential for chance findings, several mechanisms could underlie associations with placental morphological features. Placental shape, thickness, and umbilical cord placement reflect a dynamic relationship that establishes placental efficiency and impacts fetal growth [9]. Although there is limited work directly addressing these topics in ASD, some prior work suggests relationships between cord features and neurodevelopmental outcomes may be mediated by placental vascular inefficiency, evident by sparser chorionic vascular distribution or sub-optimal vascular tree branching structure [7, 49]. For example, studies have found that non-central [7] or eccentric umbilical cord insertion [49] is associated with reduced placental transport efficiency or suboptimal vasculature. In addition, though we did not see strong and consistent evidence for an association with thickness, placentas with non-central cord insertion tend to be heavier and thicker, and this may be a compensatory mechanism for reduced vascular efficiency due to deformation of the placental vasculature by non-central cord insertion [4, 5, 7, 9]. It is possible that cord eccentricity affects fetal development in utero and predisposing the fetus to later conditions [5, 31, 50]. Associations with specific placental morphology features may also provide clues as to critical periods of development or susceptibility; for example, umbilical cord eccentricity (and overall placental shape) is largely determined early within the first trimester, by approximately 11–14 weeks [51]. Future work may therefore consider placental characteristics, including features assessed here, as potential mediators of upstream exposures.

In exploratory cluster analyses, the grouping identified in our work characterizing smaller/uniform/thicker placentas is similar to that previously described as a preeclampsia placental phenotype [52]. While our findings do not suggest associations of identified clusters and child ASD-related outcomes examined here, these clusters suggest the smaller the placenta, the rounder its shape. We also showed, with the clustering approach informed by the literature, that the more eccentric the cord, the thicker the placenta. Future studies should further consider not only the vascular branching characteristics that may underlie associations with cord/morphological features observed here, but also how such groupings of placental features may relate to child developmental outcomes.

Although our study did not observe associations between placental morphological features and cognitive development as measured by the MSEL ELC, some studies have suggested links with related features and similar outcome measures. One prior study has demonstrated relationships between low-placenta-to-birthweight ratio and delayed neurodevelopment measured by the MSEL at seven time points, from 1 month to 24 months of age [19], while other studies reported links between placental inflammatory pathology (indicated by histologic chorioamnionitis) [20], placental abruption [22], placental chorionic plate diameters, disk thickness [21] and adverse child neurodevelopment according to Bayley motor and mental scales at 8 months and/or lower IQ scores at 4 and 7 years [20–22], Additionally, one study suggested positive associations between placental size (weight, surface area and placental-to-birth-weight ratio) and mental health problems in boys at 8 and 16 years of age indicated by the Rutter B2 scale [23]. An analysis of the CHARGE (Childhood Autism Risks from Genetics and the Environment) study also suggested that placental insufficiency plays a role in developmental delays, given an association between self-reported severe preeclampsia and scores on the MSEL and Vineland Adaptive Behavior Scales at ages 2 to 5 years [53]. These findings suggest a broader consideration of both the placental features examined, and the child neurodevelopmental outcomes, may be informative.

The null results observed in the present study should be considered within the context of our study population, which is enriched for ASD. It is possible that the familial background probability of ASD and ASD-related traits outweighed the influence of placental morphologic features associated with these outcomes that might be observed in other study populations. The fact that morphological differences between high autism probability and general population pregnancies have been observed in prior work [33] suggests this could be the case. Future research comparing placental morphologic features between high autism probability and general population samples is needed to clarify these questions.

This study has several strengths. First, we used prospectively collected data and had the ability to examine a range of placental features. Second, we examined associations between placental morphological measures and child outcomes in a high autism probability cohort, extending previous work. However, several limitations must also be noted. Though the sample size of the EARLI study is in line with other infant sibling studies, our analytic sample size with available data was small. Although our focus on continuous outcome measures compensated for this to some extent, most analyses yielded estimates with wide confidence intervals. Because of this, as well as the number of tests performed, results should be interpreted with caution. We also had limited ability to examine sex differences, which have been suggested in some prior work examining relationships between placental morphology and birth outcomes [10, 54]. We cannot rule out limited generalizability of our findings, which may be limited to those with relatively higher socioeconomic status, or to those with increased familial likelihood of ASD. Though our results were broadly consistent across analyses of SRS and ASD, future studies in larger samples should also consider associations with ASD diagnosis. Additionally, future work could consider other neurodevelopmental outcomes that may be influenced by placental features but that were not available in the current study. Lastly, work expanding analyses here to consider additional placental features, including vascular features and pathology, is warranted.

## Conclusions

In this high familial autism probability cohort, we found evidence for a modest association between umbilical cord eccentricity, but not other placental morphological features, and ASD-related traits. As our sample size was small, future studies are needed to confirm findings here. Given the placenta is the key interface between the mother and the developing fetus, continued work should examine how placental features may relate to ASD and other developmental outcomes.

## Supplementary Information

**Additional file 1.**

**Additional file 2.**

## Data Availability

The datasets supporting the conclusions of this article are available on request from the corresponding author. The data are not publicly available due to privacy or ethical restrictions; however, EARLI does share certain data with the National Database for Autism Research (NDAR).

## Abbreviations

EARLI: Early Autism Risk Longitudinal Investigation
ASD: Autism spectrum disorder
SRS: Social Responsiveness Scale
MSEL: Mullen Scales of Early Learning
ELC: Early Learning Composite
FGR: fetal growth restriction
BMI: body mass index
NCS: National Children’s Study
ADOS: Autism Diagnostic Observation Schedule
ADI-R: Autism Diagnostic Interview-Revised
PCA: principal components analysis
CHARGE: Childhood Autism Risks from Genetics and the Environment
