# Supplementary material for: Placental morphology in association with autism-related traits in the EARLI study
Source: BMC Pregnancy Childbirth. 2022 Jun 28;22:525. doi: 10.1186/s12884-022-04851-4 (PMC9241175; doi:10.1186/s12884-022-04851-4)
Supplement: Supplementary file 1 — Additional file 1. [file 12884_2022_4851_MOESM1_ESM.docx]

**Appendix A**

The data-driven clustering approach included 19 placenta measures that explained most of the variance based on the standardized principal components (PCs). The first three variables with highest eigenvector values from each PC were included in the k-means cluster analysis following standardization by z-scoring. In the literature-driven approach, k-means clustering analysis was conducted using 15 placenta measures based a priori knowledge and existing literature.

K-means clustering analysis is a type of unsupervised clustering method that groups subjects according to a given set of characteristics; here, placental morphology features. The optimal number of clusters (k) was determined based on the majority rule using a combination of graphic methods and statistical testing methods. The results indicating number of clusters were assessed using Clusters silhouette plots and the corresponding average silhouette width, which describes how far the sample is from the neighboring clusters; a higher value (closer to 1) indicates a better-quality clustering (i.e. far away from the neighboring clusters) and a negative value indicates the sample is in the wrong cluster. Two sensitivity analyses were conducted using the Manhattan distances and the k-medoid method, which calculate the center based on the most centrally located point in the cluster, therefore less sensitive to noise and outliers.

**Appendix Table 1. Variable selection for k-means cluster analysis**

| Variables | Variables included in PCA | Variables selected based on PCA results | Literature-driven method |
| --- | --- | --- | --- |
| **Basic placental parameters** | | | |
| Perimeter | X | X | X |
| Area | X | X | X |
| Maximum diameter | X | X | X |
| Orthogonal diameter | X | X |  |
| Area of the traced central slice | X | X |  |
| Perimeter of the traced central slice | X |  |  |
| Length of the traced central slice | X |  |  |
| Placenta weight | X | X | X |
| **Thickness parameters** | | | |
| Max thickness of traced central slice | X | X | X |
| Mean thickness of traced central slice | X |  | X |
| Standard deviation of thickness of traced central slice | X | X | X |
| **Umbilical cord parameters** | | | |
| Umbilical cord location (distance from center) | X |  | X |
| Umbilical cord eccentricity | X | X | X |
| Geometric center eccentricity | X |  | X |
| **Placental radius features** | | | |
| Minimum | X |  | X |
| Maximum | X |  | X |
| Median | X |  | X |
| Mean | X |  |  |
| Standard deviation | X | X |  |
| Skewness | X | X |  |
| Kurtosis | X | X |  |
| Standard deviation of radii with respect to mean radius from insertion point | X | X |  |
| Standard deviation of radii with respect to mean radius from geometric center | X |  |  |
| Mean of the radii obtained from insertion point | X |  |  |
| Mean of the radii obtained from centroid of disc | X |  |  |
| Radius of a circle | X |  |  |
| Radii skewness obtained from insertion point | X | X |  |
| Radii skewness obtained from geometric center | X | X |  |
| Radii kurtosis obtained from insertion point | X | X |  |
| Radii kurtosis obtained from geometric center | X | X |  |
| **Symmetry measures** | | | |
| Disc Symmetry. The center of the circle and the insertion point of the disc are placed at the origin. | X |  |  |
| Disc Symmetry. The center of the circle and the centroid of the disc are placed at the origin | X | X | X |
| Radius symmetry. The center of the circle and the insertion point of the disc are placed at the origin. | X | X | X |
| Radius symmetry. The center of the circle and the centroid of the disc are placed at the origin | X |  |  |
