# Supplementary material for: Placental morphology in association with autism-related traits in the EARLI study
Source: BMC Pregnancy Childbirth. 2022 Jun 28;22:525. doi: 10.1186/s12884-022-04851-4 (PMC9241175; doi:10.1186/s12884-022-04851-4)
Supplement: Supplementary file 2 — Additional file 2. [file 12884_2022_4851_MOESM2_ESM.docx]

eTable 1. Placental morphology measure definitions.

| **Variable** | **Units** | **Definition** |
| --- | --- | --- |
| Perimeter | cm | Placental perimeter calculated from the chorionic disk shape by counting the edge pixels and converted to cm |
| Area | cm^2^ | Placental area calculated by counting the total number of pixels occupied by the chorionic disk and converted to sq.cm |
| Radius | cm | Distance between the umbilical cord insertion point and each point on the perimeter |
| Umbilical distance from center | cm | Distance between umbilical cord insertion point and geometric center of the chorionic disk |
| Eccentricity |  |  |
| Geometric center | NA (ratio) | Ratio of the maximum radius divided by the minimum radius from the geometric center |
| Umbilical cord | NA (ratio) | Ratio of the maximum radius divided by the minimum radius from the umbilical insertion point |
| Thickness of traced central slice | cm | Thickness of the placenta as measured by the distance between each vertical pixel pair of the central placental slice |
| Placental weight | g | Placental weight at delivery after trimming extraplacental membranes and umbilical cord |
| Fetoplacental ratio | NA (ratio) | Ratio of birthweight: placental weight |

eTable 2. Correlation table for individual placental morphological features

| **Variable** | 2 | 3 | 4 | 5 | 6 | 7 | 8 | 9 | 10 | 11 | |  |
| --- | --- | --- | --- | --- | --- | --- | --- | --- | --- | --- | --- | --- |
| 1. Perimeter | 0.94 | 0.35 | 0.50 | 0.51 | -0.10 | 0.67 | -0.09 | -0.14 | -0.40 | 0.63 | |  |
| 2. Area | 1.00 | 0.39 | 0.49 | 0.58 | -0.09 | 0.47 | -0.10 | -0.14 | -0.36 | 0.68 | |  |
| 3. Radius, Minimum |  | 1.00 | -0.52 | -0.42 | -0.9 | 0.16 | -0.73 | -0.04 | -0.12 | 0.18 | |  |
| 4. Radius, Maximum |  |  | 1.00 | 0.77 | 0.76 | 0.36 | 0.56 | -0.08 | -0.22 | 0.36 | |  |
| 5. Radius, Median |  |  |  | 1.00 | 0.62 | 0.14 | 0.54 | -0.07 | -0.18 | 0.49 | |  |
| 6. Umbilical cord distance from center |  |  |  |  | 1.00 | -0.12 | 0.72 | 0.01 | 0.04 | 0.04 | |  |
| 7. Eccentricity from geometric center |  |  |  |  |  | 1.00 | -0.08 | -0.02 | -0.27 | 0.24 | |  |
| 8.Eccentricity from cord insertion point |  |  |  |  |  |  | 1.00 | 0.04 | 0.05 | 0.01 | |  |
| 9. Thickness of traced central slice, Maximum |  |  |  |  |  |  |  | 1.00 | 0.81 | 0.35 | |  |
| 10. Thickness of traced central slice, Mean |  |  |  |  |  |  |  |  | 1.00 | | 0.21 |  |
| 11. Placental weight |  |  |  |  |  |  |  |  |  | | 1.00 |  |

eTable 3. Basic characteristics of the EARLI study population by analysis inclusion

|  | **Included**^a^ **(N=101)** | **Not Included**^b^ **(N=155)** |
| --- | --- | --- |
| **Maternal characteristics** | **Mean (SD) or n(%)** | **Mean (SD) or n(%)** |
| Maternal Age | 34.6 (4.18) | 33.2 (5.10) |
| Maternal Race/Ethnicity |  |  |
| Non-Hispanic White | 62 (61.4%) | 75 (48.4%) |
| Hispanic | 11 (10.9%) | 31 (20.0%) |
| Other/Missing | 28 (27.7%) | 49 (31.6%) |
| Maternal Education Level |  |  |
| Some college or below | 33 (32.7%) | 71 (45.8%) |
| College degree | 31 (30.7%) | 41 (26.5%) |
| Graduate School or above | 35 (34.7%) | 35 (22.6%) |
| Maternal Birthplace |  |  |
| US | 84 (83.2%) | 1 (0.6%) |
| Outside U.S. | 16 (15.8%) | 0 (0%) |
| Missing | 1 (1.0%) | 154 (99.4%) |
| Maternal Pre-pregnancy BMI | 27.1 (6.67) | 28.1 (6.87) |
| Household Income |  |  |
| Less than $50,000 | 25 (24.8%) | 0 (0%) |
| $50,000 to $99,999 | 26 (25.7%) | 0 (0%) |
| $100,000 or more | 47 (46.5%) | 1 (0.6%) |
| Missing | 3 (3.0%) | 154 (99.4%) |
| **Child characteristics** | **Mean (SD) or n(%)** | **Mean (SD) or n(%)** |
| Child Sex |  |  |
| Female | 46 (45.5%) | 70 (45.2%) |
| Male | 55 (54.5%) | 85 (54.8%) |
| Child Birthweight |  |  |
| Normal birthweight | 96 (95.0%) | 115 (74.2%) |
| Low birthweight (<2500g) | 4 (4.0%) | 13 (8.4%) |
| Missing | 1 (1.0%) | 27 (17.4%) |
| Gestational Age |  |  |
| Preterm | 7 (6.9%) | 22 (14.2%) |
| Full-term | 93 (92.1%) | 128 (82.6%) |
| Post-term | 1 (1.0%) | 5 (3.2%) |
| Delivery Mode |  |  |
| Vaginal Birth | 61 (60.4%) | 0 (0%) |
| C-section | 38 (37.6%) | 1 (0.6%) |
| Missing | 2 (2.0%) | 154 (99.4%) |
| SRS Total Raw Score at 36mos | 32.9 (27.1) | 42.0 (NA) |
| MSEL ELC Score at 36mos | 99.2 (20.7) | 122 (NA) |
| ASD Diagnosis at 36mos |  |  |
| No ASD | 80 (79.2%) | 1 (0.6%) |
| With ASD | 20 (19.8%) | 0 (0%) |
| Missing | 1 (1.0%) | 154 (99.4%) |

^a^ Maternal Pre-pregnancy BMI missing n=4. SRS Total Raw Score at 36mos missing n=17. MSEL ELC Score at 36mos missing n=5.

^b^ Maternal age missing n=10. Maternal Pre-pregnancy BMI missing n=11. SRS Total Raw Score at 36mos missing n=154. MSEL ELC Score at 36mos missing n=154

eTable 4. Crude and adjusted associations (ß estimates and 95% confidence intervals) between placental morphological features standardized for overall placental size and child raw total SRS scores

|  |  | **Crude** | **Model 1**^a^ | **Model 2**^b^ |
| --- | --- | --- | --- | --- |
| **Variable** | **n** | ß (95% CI) | ß (95% CI) | ß (95% CI) |
| Radius / Area |  |  |  |  |
| Minimum / Area | 84 | -4.09 (-10.0, 1.8) | -2.81 (-8.5, 2.8) | -2.79 (-8.5, 2.9) |
| Maximum / Area | 84 | 4.02 (-1.8, 9.8) | 0.70 (-5.1, 6.5) | 0.87 (-5.1, 6.8) |
| Median / Area | 84 | 2.69 (-3.4, 8.7) | 0.47 (-5.5, 6.4) | 0.64 (-5.5, 6.7) |
| Umbilical cord distance from center/ Area | 84 | 3.88 (-2.0, 9.7) | 1.28 (-4.5, 7.1) | 1.34 (-4.5, 7.2) |
|  |  |  |  |  |

^a^ Model 1 adjusted for child sex, maternal age, maternal education level, maternal pre-pregnancy BMI.

^b^ Model 2 adjusted for all covariates from Model 1 and additionally adjusted for child gestational age. Additional adjustment for maternal race/ethnicity did not materially alter results.

eTable 5. Crude and adjusted associations (ß estimates and 95% confidence intervals) between placental morphological features standardized for overall placental size and child MSEL ELC scores

|  |  | **Crude** | **Model 1**^b^ | **Model 2**^c^ |
| --- | --- | --- | --- | --- |
| **Variable**^a^ | **n** | ß (95% CI) | ß (95% CI) | ß (95% CI) |
| Radius / Area |  |  |  |  |
| Minimum / Area | 96 | -0.00 (-4.2, 4.2) | -0.44 (-4.4, 3.5) | -0.48 (-4.4, 3.5) |
| Maximum / Area | 96 | 0.72 (-3.5, 4.9) | 2.49 (-1.5, 6.5) | 2.29 (-1.8, 6.4) |
| Median / Area | 96 | 0.75 (-3.5, 5.0) | 0.77 (-3.2, 4.8) | 0.63 (-3.4, 4.7) |
| Umbilical cord distance from center/ Area | 96 | 0.33 (-3.9, 4.5) | 1.51 (-2.5, 5.5) | 1.40 (-2.6, 5.4) |
|  |  |  |  |  |

^a^ Model 1 adjusted for child sex, maternal age, maternal education level, maternal pre-pregnancy BMI.

^b^ Model 2 adjusted for all covariates from Model 1 and additionally adjusted for child gestational age. Additional adjustment for maternal race/ethnicity did not materially alter results.

eTable 6. Crude and adjusted associations (ß estimates and 95% confidence intervals) between the individual placental variable in quartiles and offspring outcomes

|  | **SRS Raw Total Scores** | | | **MSEL ELC Scores** | | |
| --- | --- | --- | --- | --- | --- | --- |
|  |  | **Crude** | **Adjusted**^a^ |  | **Crude** | **Adjusted**^a^ |
|  | n | ß (95% CI) | ß (95% CI) | n | ß (95% CI) | ß (95% CI) |
| Perimeter (cm) |  |  |  |  |  |  |
| Lowest quartile | 23 | -4.11 (-18.41, 10.18) | -4.58 (-18.37, 9.21) | 25 | 2.08 (-8.11, 12.27) | 2.22 (-7.33, 11.77) |
| Interquartile range | 39 | *1.00 (Reference)* | *1.00 (Reference)* | 49 | *1.00 (Reference)* | *1.00 (Reference)* |
| Highest quartile | 22 | -3.66 (-18.16, 10.84) | 1.85 (-12.47, 16.16) | 22 | -1.37 (-12.01, 9.27) | -3.65 (-13.98, 6.69) |
| Area (cm**^2^)** |  |  |  |  |  |  |
| Lowest quartile | 23 | -1.43 (-15.63, 12.76) | -5.35 (-19.10, 8.40) | 25 | 1.00 (-9.14, 11.13) | 3.66 (-5.91, 13.24) |
| Interquartile range | 40 | *1.00 (Reference)* | *1.00 (Reference)* | 49 | *1.00 (Reference)* | *1.00 (Reference)* |
| Highest quartile | 21 | 5.33 (-9.29, 19.96) | 3.21 (-11.07, 17.48) | 22 | -5.57 (-16.15, 5.01) | -1.97 (-12.12, 8.18) |
| Minimum radius (cm) |  |  |  |  |  |  |
| Lowest quartile | 19 | -1.57 (-16.46, 13.32) | -1.20 (-16.07, 13.67) | 23 | 6.61 (-3.78, 17.00) | 4.00 (-5.97, 13.96) |
| Interquartile range | 43 | *1.00 (Reference)* | *1.00 (Reference)* | 49 | *1.00 (Reference)* | *1.00 (Reference)* |
| Highest quartile | 22 | -8.28 (-22.45, 5.89) | -1.25 (-15.38, 12.89) | 24 | 4.86 (-5.38, 15.11) | -0.88 (-10.86, 9.11) |
| Maximum radius (cm) |  |  |  |  |  |  |
| Lowest quartile | 23 | 6.18 (-7.64, 20.00) | 4.71 (-8.67, 18.10) | 25 | -8.12 (-18.12, 1.88) | -6.69 (-16.19, 2.81) |
| Interquartile range | 39 | *1.00 (Reference)* | *1.00 (Reference)* | 47 | *1.00 (Reference)* | *1.00 (Reference)* |
| Highest quartile | 22 | 17.40 (3.38, 31.42) | 12.59 (-1.76, 26.93) | 24 | -10.79 (-20.92, -0.66) | -6.10 (-16.21, 4.01) |
| Median radius (cm) |  |  |  |  |  |  |
| Lowest quartile | 23 | -0.72 (-14.86, 13.42) | -4.23 (-18.29, 9.83) | 24 | 3.75 (-6.52, 14.02) | 3.90 (-5.68, 13.48) |
| Interquartile range | 41 | *1.00 (Reference)* | *1.00 (Reference)* | 50 | *1.00 (Reference)* | *1.00 (Reference)* |
| Highest quartile | 20 | 5.60 (-9.21, 20.40) | 5.54 (-8.62, 19.70) | 22 | -1.09 (-11.68, 9.50) | -4.10 (-14.16, 5.96) |
| Minimum radius/ Area |  |  |  |  |  |  |
| Lowest quartile | 19 | 9.52 (-5.3, 24.4) | 4.83 (-10.7, 20.3) | 24 | 0.92 (-9.5, 11.3) | 1.38 (-8.7, 11.4) |
| Interquartile range | 43 | *1.00 (Reference)* | *1.00 (Reference)* | 47 | *1.00 (Reference)* | *1.00 (Reference)* |
| Highest quartile | 22 | 4.60 (-9.5, 18.7) | 2.59 (-11.1, 16.2) | 25 | -3.35 (-13.6, 6.9) | -3.25 (-12.8, 6.3) |
| Maximum radius/ Area |  |  |  |  |  |  |
| Lowest quartile | 22 | -5.94 (-20.2, 8.3) | 4.32 (-10.2, 18.8) | 23 | 2.64 (-7.8, 13.1) | -2.98 (-13.0, 7.0) |
| Interquartile range | 42 | *1.00 (Reference)* | *1.00 (Reference)* | 48 | *1.00 (Reference)* | *1.00 (Reference)* |
| Highest quartile | 20 | 1.38 (-13.3, 16.1) | 2.57 (-11.5, 16.7) | 25 | 5.09 (-5.1, 15.3) | 5.04 (-4.5, 14.5) |
| Median radius/ Area |  |  |  |  |  |  |
| Lowest quartile | 22 | 2.90 (-11.3, 17.1) | 7.31 (-6.5, 21.1) | 23 | 2.93 (-7.5, 13.4) | 2.16 (-7.7, 12.0) |
| Interquartile range | 43 | *1.00 (Reference)* | *1.00 (Reference)* | 49 | *1.00 (Reference)* | *1.00 (Reference)* |
| Highest quartile | 19 | 7.18 (-7.8, 22.1) | 7.77 (-6.7, 22.2) | 24 | 4.96 (-5.3, 15.3) | 4.00 (-5.8, 13.7) |
| Umbilical distance from center (cm)* | | |  |  |  |  |
| Lowest quartile | 23 | 0.92 (-12.77, 14.60) | 5.44 (-7.79, 18.68) | 24 | -3.38 (-13.72, 6.97) | -6.63 (-16.28, 3.01) |
| Interquartile range | 42 | *1.00 (Reference)* | *1.00 (Reference)* | 48 | *1.00 (Reference)* | *1.00 (Reference)* |
| Highest quartile | 19 | 16.52 (1.94, 31.11) | 13.55 (-0.70, 27.81) | 24 | -3.88 (-14.22, 6.47) | -3.31 (-12.97, 6.35) |
| Umbilical distance from center/ Area* | | |  |  |  |  |
| Lowest quartile | 23 | -10.74 (-24.7, 3.3) | -3.28 (-17.2, 10.7) | 24 | 2.41 (-7.9, 12.7) | -3.13 (-13.0, 6.8) |
| Interquartile range | 41 | *1.00 (Reference)* | *1.00 (Reference)* | 49 | *1.00 (Reference)* | *1.00 (Reference)* |
| Highest quartile | 20 | -3.78 (-18.4, 10.9) | -3.64 (-18.0, 10.7) | 23 | 3.12 (-7.4, 13.6) | 1.36 (-8.6, 11.4) |
| Geometric center eccentricity | | |  |  |  |  |
| Lowest quartile | 19 | 1.28 (-13.75, 16.31) | 2.52 (-11.79, 16.83) | 22 | 2.08 (-8.43, 12.60) | 2.68 (-7.17, 12.54) |
| Interquartile range | 42 | *1.00 (Reference)* | *1.00 (Reference)* | 51 | *1.00 (Reference)* | *1.00 (Reference)* |
| Highest quartile | 23 | 4.55 (-9.55, 18.66) | 5.66 (-7.82, 19.14) | 23 | 6.16 (-4.20, 16.51) | 6.72 (-2.89, 16.32) |
| Umbilical cord eccentricity | | |  |  |  |  |
| Lowest quartile | 22 | -0.44 (-14.72, 13.83) | 2.41 (-11.27, 16.09) | 25 | -3.29 (-13.55, 6.96) | -5.34 (-14.92, 4.24) |
| Interquartile range | 43 | *1.00 (Reference)* | *1.00 (Reference)* | 47 | *1.00 (Reference)* | *1.00 (Reference)* |
| Highest quartile | 19 | 2.51 (-12.49, 17.52) | 2.32 (-12.15, 16.79) | 24 | 0.61 (-9.78, 11.00) | -0.58 (-10.32, 9.16) |
| Maximum thickness of traced central slice (cm) | | | |  |  |  |
| Lowest quartile | 20 | -3.62 (-18.64, 11.40) | -7.18 (-22.10, 7.74) | 20 | -6.94 (-17.81, 3.93) | -4.22 (-14.98, 6.55) |
| Interquartile range | 41 | *1.00 (Reference)* | *1.00 (Reference)* | 49 | *1.00 (Reference)* | *1.00 (Reference)* |
| Highest quartile | 20 | 3.58 (-11.44, 18.60) | 1.84 (-12.62, 16.31) | 24 | -10.17 (-20.37, 0.04) | -6.33 (-16.30, 3.64) |
| Mean thickness of traced central slice (cm) | | |  |  |  |  |
| Lowest quartile | 19 | 4.86 (-10.23, 19.95) | 6.05 (-8.79, 20.88) | 21 | -7.19 (-17.84, 3.46) | -6.91 (-17.33, 3.51) |
| Interquartile range | 41 | *1.00 (Reference)* | *1.00 (Reference)* | 49 | *1.00 (Reference)* | *1.00 (Reference)* |
| Highest quartile | 21 | 12.12 (-2.47, 26.71) | 11.59 (-2.63, 25.82) | 23 | -11.07 (-21.39, -0.75) | -7.88 (-17.76, 2.01) |
| Placental weight (g) |  |  |  |  |  |  |
| Lowest quartile | 20 | -1.75 (-16.63, 13.14) | -2.32 (-16.42, 11.78) | 23 | 1.31 (-9.33, 11.95) | 1.15 (-8.79, 11.09) |
| Interquartile range | 39 | *1.00 (Reference)* | *1.00 (Reference)* | 45 | *1.00 (Reference)* | *1.00 (Reference)* |
| Highest quartile | 20 | 6.10 (-8.78, 20.99) | 11.29 (-3.31, 25.90) | 21 | -4.50 (-15.47, 6.48) | -6.20 (-16.82, 4.41) |
| Birthweight: Placental weight Ratio | | |  |  |  |  |
| Lowest quartile | 18 | 13.29 (-1.68, 28.27) | 20.62 (5.28, 35.97) | 20 | 0.10 (-11.05, 11.25) | -0.72 (-12.62, 11.19) |
| Interquartile range | 43 | *1.00 (Reference)* | *1.00 (Reference)* | 45 | *1.00 (Reference)* | *1.00 (Reference)* |
| Highest quartile | 18 | 2.41 (-12.57, 17.38) | 0.70 (-13.25, 14.66) | 23 | -2.07 (-12.70, 8.56) | -0.50 (-10.45, 9.44) |

^a^ Model adjusted for child sex, maternal age, maternal education level, maternal pre-pregnancy BMI, child gestational age, maternal race/ethnicity

eTable 7. Crude and adjusted associations (OR and 95%CI) between individual placental variables and offspring ASD diagnosis

| **Variable**^a^ | **N**^b^ | **Crude** | **Model 1**^c^ | **Model 2**^d^ |
| --- | --- | --- | --- | --- |
| Perimeter (cm) | 100 | 0.96 (0.89, 1.03) | 0.97 (0.89, 1.06) | 0.97 (0.89, 1.07) |
| Area (cm^2^) | 100 | 0.99 (0.98, 1.01) | 1.00 (0.98, 1.01) | 1.00 (0.98, 1.01) |
| Radius (cm) |  |  |  |  |
| Minimum | 100 | 0.88 (0.69, 1.11) | 0.92 (0.70, 1.20) | 0.92 (0.70, 1.21) |
| Maximum | 100 | 1.02 (0.82, 1.26) | 0.97 (0.77, 1.23) | 0.98 (0.78, 1.24) |
| Median | 100 | 0.97 (0.64, 1.47) | 1.04 (0.67, 1.63) | 1.08 (0.68, 1.72) |
| Radius / Area |  |  |  |  |
| Minimum | 100 | 0.87 (0.54, 1.43) | 0.92 (0.54, 1.58) | 0.91 (0.53, 1.56) |
| Maximum | 100 | 1.28 (0.79, 2.07) | 1.09 (0.62, 1.91) | 1.06 (0.60, 1.88) |
| Median | 100 | 1.30 (0.80, 2.10) | 1.30 (0.74, 2.30) | 1.28 (0.72, 2.27) |
| Umbilical distance from center (cm) | 100 | 1.08 (0.85, 1.37) | 1.01 (0.78, 1.30) | 1.01 (0.78, 1.31) |
| Umbilical distance from center / Area | 100 | 1.17 (0.72, 1.90) | 1.03 (0.60, 1.77) | 1.02 (0.59, 1.76) |
| Eccentricity |  |  |  |  |
| Geometric center | 100 | 1.00 (0.14, 7.2) | 1.31 (0.11, 15.93) | 1.40 (0.11, 17.14) |
| Umbilical cord | 100 | 1.13 (1.01, 1.25) | 1.12 (0.99, 1.26) | 1.13 (1.00, 1.27) |
| Thickness of traced central slice (cm) |  |  |  |  |
| Maximum | 97 | 1.13 (0.26, 4.85) | 1.04 (0.20, 5.24) | 1.20 (0.23, 6.33) |
| Mean | 97 | 1.08 (0.16, 7.12) | 1.29 (0.15, 10.9) | 1.50 (0.17, 13.09) |
| Thickness of traced central slice (cm) (Z score) |  |  |  |  |
| Maximum | 97 | 1.04 (0.64, 1.70) | 1.01 (0.59, 1.74) | 1.06 (0.61, 1.85) |
| Mean | 97 | 1.02 (0.63, 1.67) | 0.07 (0.06, 0.81) | 1.11 (0.63, 1.95) |
| Placental weight (g) | 93 | 1.00 (0.99, 1.00) | 1.00 (0.99, 1.01) | 1.00 (0.99, 1.01) |
| Birthweight: Placental weight ratio | 92 | 1.60 (0.933, 2.74) | 1.22 (0.64, 2.30) | 1.24 (0.66, 2.35) |

^a^ Placental eccentricity is the ratio of the maximum radius divided by the minimum radius from the geometric center or the umbilical insertion point; a round placenta with a centrally inserted umbilical cord would have a cord eccentricity of 1.

^b^ Includes 20 ASD cases.

^c^ Model 1 adjusted for child sex, maternal age, maternal education level, maternal pre-pregnancy BMI.

^d^ Model 2 adjusted for all covariates from Model 1 and additionally adjusted for child gestational age. Additional adjustment for maternal race/ethnicity did not materially alter results.

eTable 8. Associations (ß estimates and 95% confidence intervals) between cluster-derived placental characteristics and continuous ASD-related outcomes according to two clustering approaches

|  |  | **Crude Model** | **Model 1**^b^ | **Model 2**^c^ |  |
| --- | --- | --- | --- | --- | --- |
|  | n^a^ | ß (95% CI) | ß (95% CI) | ß (95% CI) |  |
| Data-driven clustering approach | | | | |  |
| SRS total raw score | 77 | 5.47 (-7.31, 18.24) | 0.48 (-12.52, 13.47) | 0.91 (-12.21, 14.03) | |
| MSEL ELC score | 87 | -1.88 (-11.01, 7.25) | 0.05 (-8.76, 8.86) | -0.36 (-9.25, 8.54) |  |
| Literature-driven clustering approach | | | | |  |
| SRS total raw score | 77 | 7.13 (-5.52, 19.77) | 3.63 (-8.51, 15.77) | 2.94 (-9.54, 15.42) |  |
| MSEL ELC score | 87 | -0.37 (-9.65, 8.90) | 3.04 (-5.70, 11.79) | 3.51 (-5.32, 12.34) |  |

^a^ Missing value for maternal pre-pregnancy BMI (n=4), maternal education level (n= 2), maternal race/ethnicity (n=1) were imputed with median value.

^b^ Model 1 adjusted for child sex, maternal age, maternal education level, maternal pre-pregnancy BMI.

^c^ Model 2 adjusted for all covariates from Model 1 and additionally adjusted for child gestational age. Additional adjustment for maternal race/ethnicity did not materially alter results.

eFigure 1. Data-driven approach: Standardized placental attributes distribution by cluster assignment.


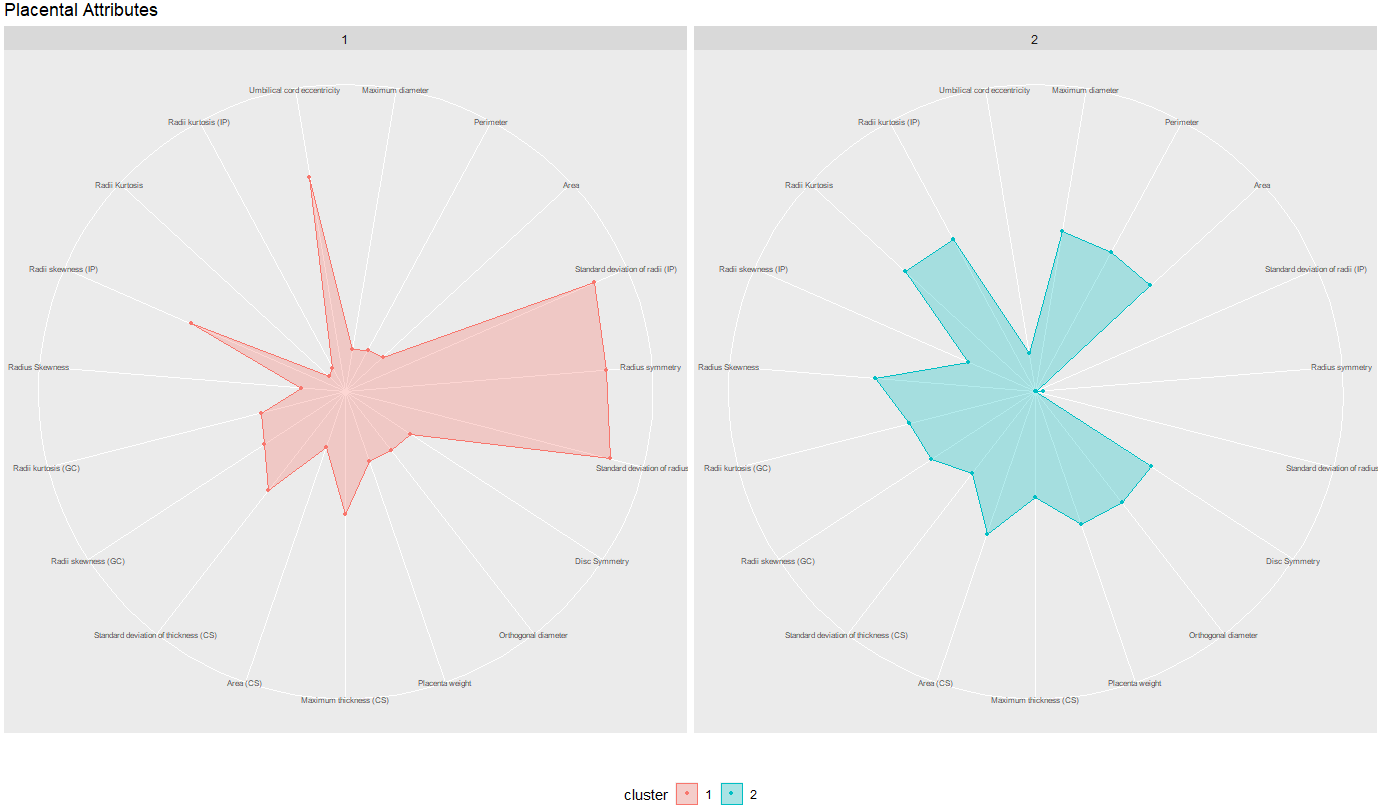


**Figure legend.** The radar plots show the average value of each standardized placental attributes in each of the identified cluster (the closer to the center, the lower the value). Around the perimeter of each plot, placental variables included in the cluster analysis are shown. These variables are also listed in Table Appendix Table 1. The data-driven approach identified two clusters. The first cluster contained individuals generally characterized by smaller, more uniform, but thicker placentas, while the second cluster contained those characterized by larger, thinner placentas and a less centrally inserted umbilical cord.

eFigure 2. Literature driven approach: Standardized placental attributes distribution by cluster assignment.


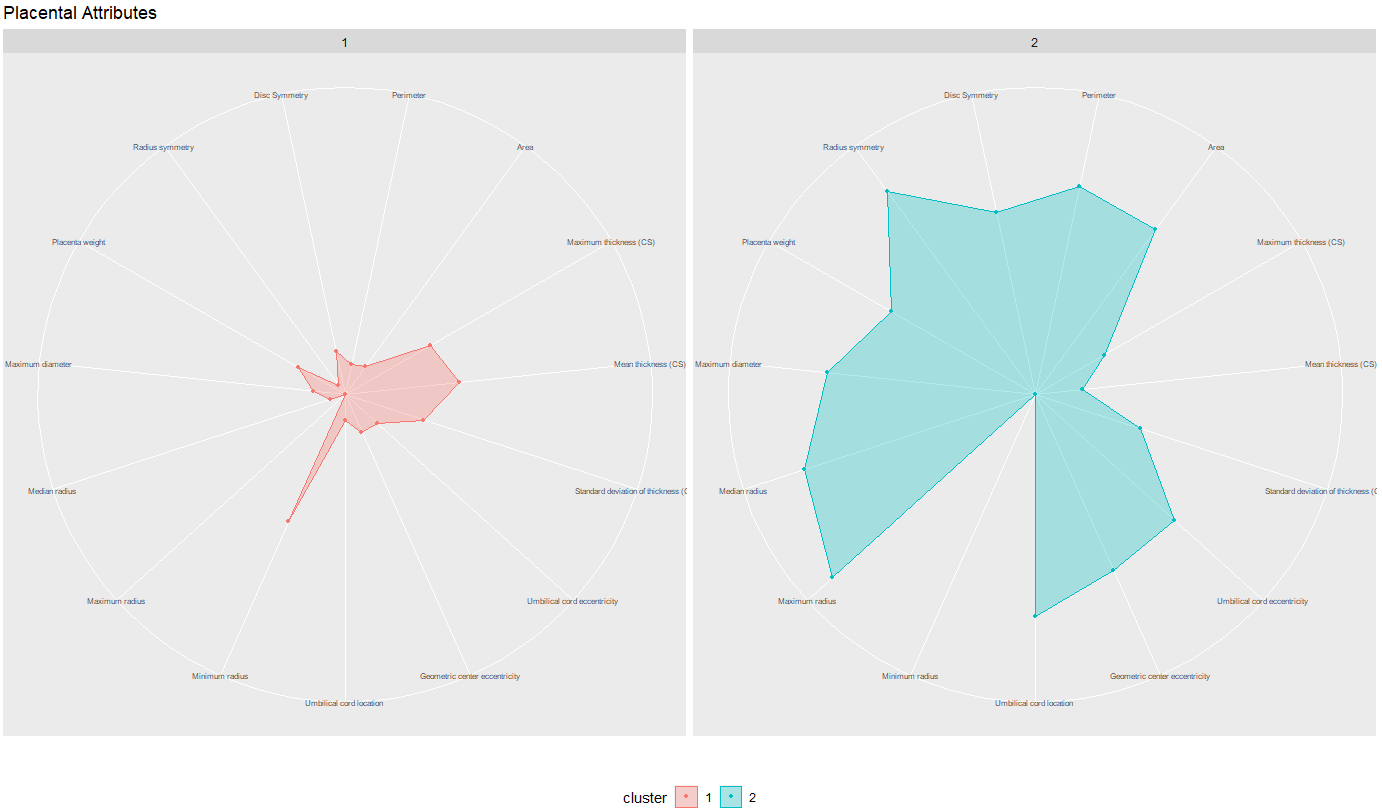


**Figure legend.** The radar plots show the average value of each standardized placental attributes in each of the identified cluster (the closer to the center, the lower the value). Around the perimeter of each plot, placental variables included in the cluster analysis are shown. These variables are also listed in Table Appendix Table 1. The literature driven approach identified two clusters. The first cluster contained individuals generally characterized by smaller, thicker placentas with a less centrally inserted umbilical cord, while the second cluster contained those characterized by larger, thinner, more uniform placentas.
